# Elucidation of the MicroRNA Transcriptome in Western Corn Rootworm Reveals Its Dynamic and Evolutionary Complexity

**DOI:** 10.1016/j.gpb.2019.03.008

**Published:** 2021-02-17

**Authors:** Xiaozeng Yang, Elane Fishilevich, Marcelo A. German, Premchand Gandra, Robert E. McEwan, André Billion, Eileen Knorr, Andreas Vilcinskas, Kenneth E. Narva

**Affiliations:** 1 Beijing Agro-biotechnology Research Center, Beijing Academy of Agriculture and Forestry Sciences, Beijing 100097, China; 2 Corteva Agriscience, Agriculture Division of DowDuPont, Indianapolis, IN 46268, USA; 3 University of Nebraska-Lincoln, Department of Entomology, Lincoln, NE 68583, USA; 4 Fraunhofer Institute for Molecular Biology and Applied Ecology, Department of Bioresources, Giessen 35394, Germany

**Keywords:** Western corn rootworm, Small RNA, MicroRNA, Parallel Analysis of RNA Ends, PIWI-interacting RNA

## Abstract

*Diabrotica virgifera virgifera* (**western corn rootworm**, WCR) is one of the most destructive agricultural insect pests in North America. It is highly adaptive to environmental stimuli and crop protection technologies. However, little is known about the underlying genetic basis of WCR behavior and adaptation. More specifically, the involvement of **small RNAs** (sRNAs), especially microRNAs (miRNAs), a class of endogenous small non-coding RNAs that regulate various biological processes, has not been examined, and the datasets of putative sRNA sequences have not previously been generated for WCR. To achieve a comprehensive collection of sRNA transcriptomes in WCR, we constructed, sequenced, and analyzed sRNA libraries from different life stages of WCR and northern corn rootworm (NCR), and identified 101 conserved precursor miRNAs (pre-miRNAs) in WCR and other Arthropoda. We also identified 277 corn rootworm specific pre-miRNAs. Systematic analyses of sRNA populations in WCR revealed that its sRNA transcriptome, which includes **PIWI-interacting RNAs** (piRNAs) and miRNAs, undergoes a dynamic change throughout insect development. Phylogenetic analysis of miRNA datasets from model species reveals that a large pool of species-specific miRNAs exists in corn rootworm; these are potentially evolutionarily transient. Comparisons of WCR miRNA clusters to other insect species highlight conserved miRNA-regulated processes that are common to insects. **Parallel Analysis of RNA Ends** (PARE) also uncovered potential miRNA-guided cleavage sites in WCR. Overall, this study provides a new resource for studying the sRNA transcriptome and miRNA-mediated gene regulation in WCR and other Coleopteran insects.

## Introduction

Corn rootworm, is a pest complex that significantly affects corn yield in the United States, and more recently in Europe [1], [2]. Often referred to as a billion dollar pest [3], its damage is of major economic concern for corn growers in the US corn belt. The key corn rootworm species are western corn rootworm (WCR; *Diabrotica virgifera virgifera*), northern corn rootworm (NCR; *D. barberi*), and southern corn rootworm (SCR; *D. undecimpunctata howardi*). Both adult and larval WCR can damage corn plants. The adults feed on corn silks, kernels, tassels, and foliage [4]. The larval stages cause the most significant damage by feeding on corn roots in late spring and early summer [5]. The damage from WCR larvae can manifest in loss of the corn root mass [5], that may then result in decreased water and nutrient uptake, plant lodging and significant loss of grain yield [5], [6].

WCR and NCR have only one generation per year and their eggs may overwinter in diapause in the soil. While crop rotation with soybeans has been a major and effective strategy for rootworm control, WCR have evolved resistance to crop rotation through prolonged diapause [7] or attaining the propensity to lay eggs in soybean fields [7]. Moreover, insecticidal *Bacillus thuringiensis* (Bt) proteins, which include Cry3 family proteins and Cry34/35Ab1 and control corn rootworm, are beginning to show indications of resistance, either by confirmed field-evolved resistance as in the case of Cry3Bb1 and mCry3A or incomplete resistance as in the case of Cry34/35Ab1 [8], [9]. However, little is known about the underlying genetic basis of WCR behavior and adaptation, especially the study of small RNAs (sRNAs). Currently, several studies suggest that microRNAs (miRNAs) are differentially expressed in Bt-resistant insects and therefore may be involved in Bt resistance [10], [11].

The discovery of highly prevalent sRNAs that regulate diverse spatial and temporal biological functions [12] is one of the most exciting biological findings in the last two decades. PIWI-interacting RNAs (piRNAs), 26–33 nucleotides (nt) in size, are the largest class of small non-coding RNA molecules, and are only expressed in animal cells and interact with piwi proteins to form RNA-protein complexes [13]. The piRNA complexes have been linked to both epigenetic and post-transcriptional gene silencing [14]. Among sRNAs, miRNAs are another important class of 20–24 nt sRNAs in eukaryotes. The biogenesis pathway of miRNAs has been studied extensively. Primary transcripts of miRNAs (pri-miRNAs) are transcribed by RNA polymerase II, and can self-fold into stem-loop secondary structures that constitute precursor miRNAs (pre-miRNAs) [12]. Pre-miRNAs futher produce double-stranded RNAs 20–24 nt in length [12], [15]. Only one strand of the double-stranded RNA, the mature miRNA, is loaded into the RNA-induced silencing complex (RISC) to guide post-transcriptional gene regulation by inhibiting the translation of their target mRNAs (mainly in animals) [12] or cleaving their target mRNAs (mainly in plants) [15].

Since their discovery in the nematode *Caenorhabditis elegans* [16], [17], numerous studies and methods have aimed to identify miRNAs in different species [18], [19]. Propelled by next-generation sequencing technologies, deep sampling of size-fractionated low-molecular-weight RNA libraries, coupled with bioinformatics mining, has become a popular approach to identify miRNAs in diverse insect species [20], [21]. The release version 21 of miRBase annotation contains approximately 3200 precursors and 4000 mature miRNAs from 32 Arthropoda species [22]. Meanwhile, more and more studies have firmly established that miRNAs are involved in multiple regulatory circuits to modulate gene expression [12], [23]. The participation of miRNAs in governing insect development and lifecycle as well as in stimulus and stress responses is well documented [24]. It was also reported that miRNAs are involved in insecticide resistance [10].

In this study, we sought to generate a comprehensive collection of sRNAs in WCR. We employed a method to identify pre-miRNAs based on a model of miRNA biogenesis to retrieve miRNA-related information from both the genomic sequences and deep-sequenced sRNA libraries [25]. Using this method, with an in-house draft genome of WCR, we constructed and parsed 18 sRNA libraries from 6 life stages of WCR and two libraries from two life stages of NCR, respectively, and we identified 101 conserved and 277 corn rootworm specific and novel pre-miRNAs. Further, we found that the abundance of the two main groups of sRNAs (miRNAs and piRNAs), varies among different life stages in WCR. A systematic analysis of conservation with model species revealed that corn rootworm specific miRNAs undergo a rapid selection, while most miRNAs are highly expressed only in specific life stages. miRNA cluster analyses not only suggested that clustered miRNAs are regulated by the same *cis*-elements and transcription factors but also uncovered the evolutionary changes of even conserved clusters in different species. Taken together, these systematic analyses revealed the dynamic and evolutionary complexity of the sRNA transcriptome in WCR. Also, to sample mRNA targets of miRNAs in WCR, we constructed two Parallel Analysis of RNA Ends (PARE) libraries and uncovered potential miRNA-guided cleavage sites within WCR transcriptome. These data provide new insights into the potential functions and evolution of sRNAs, especially miRNAs, in WCR, and they represent a critical and rich resource for facilitating sRNA research and applications in WCR.

## Results

### Dynamic changes in sRNA transcriptome during rootworm development

**Figure 1:**
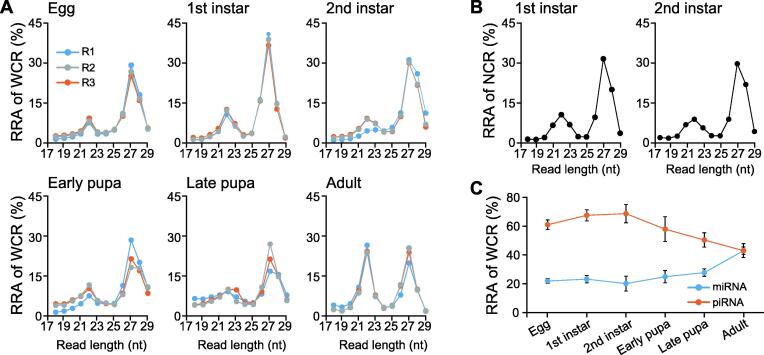
**Dynamic changes in miRNA and piRNA populations during the lifecycle of WCR and NCR** **A.** sRNA relative abundance and length distribution in six life stages of WCR. For each life stage, there are three biological replicates. **B.** sRNA relative abundance and length distribution in 1st and 2nd instar of NCR. **C.** Relative abundance of miRNAs (21–24 nt) and piRNAs (26–29 nt) in six life stages of WCR. Error bars indicate standard deviations in each life stage. WCR, western corn rootworm; NCR, northern corn rootworm; RRA, read relative abundance; miRNA, microRNA; piRNA, PIWI-interacting RNA.

To systematically profile the sRNA transcriptome in corn rootworm, we first sequenced 18 sRNA libraries spanning six life stages, from egg to adult WCR (Figure 1A, Figures S1 and S2; Table S1). Interestingly, RNA samples from 1st and 2nd instar larvae have the most abundant sRNA reads after normalization (Table S1), suggesting that more sRNAs are involved in biological processes during early instar stages as compared to later stages. For that reason, we also sequenced sRNAs of 1st and 2nd instar (Table S2) northern corn rootworm (NCR). Together, a collection of 20 samples harboring over 230 million reads was utilized to profile the sRNA signatures in corn rootworm.

The length distribution of each of the 20 sRNA libraries showed a peak at 22 nt (Figure 1A and B), reflecting the abundance of miRNAs. We also found a prominent sRNA peak at 27 nt. Recent research [13], [14] indicates that piRNAs, differing from miRNAs in size (26–29 nt instead of 21–24 nt), are the largest class of small non-coding RNA molecules in animal cells. Thus, the 27-nt peaks in all samples most likely represent piRNAs. Since piRNAs lack sequence conservation and are characterized by greater sequence diversity [13], [14], we further examined uniqueness for sRNA reads in that size category (27 nt) and found a higher relative abundance of unique sequences (Figure S2A and B), which further supports the 27-nt peak representing mainly piRNAs.

Another notable feature of the rootworm sRNA transcriptome distribution is that the relative abundances of 22-nt and 27-nt peaks are different across life stages. For example, 22-nt peaks range from 10% (egg) to 25% (adult) while the 27-nt peaks range from 40% (1st instar larva) to less than 25% (adult) (Figure 1A). To further examine the differences between these classes of sRNAs, we considered 21–24 nt as miRNAs and 26–29 nt as piRNAs. Following this assumption, a trend appeared evidently: in early life stages such as egg and larva, piRNAs are dominant compared to miRNAs, while in later life stages (pupa and adult) their abundance declines, while the abundance of miRNAs gradually increases during development (Figure 1C).

### Comprehensive identification of pre-miRNAs

**Figure 2:**
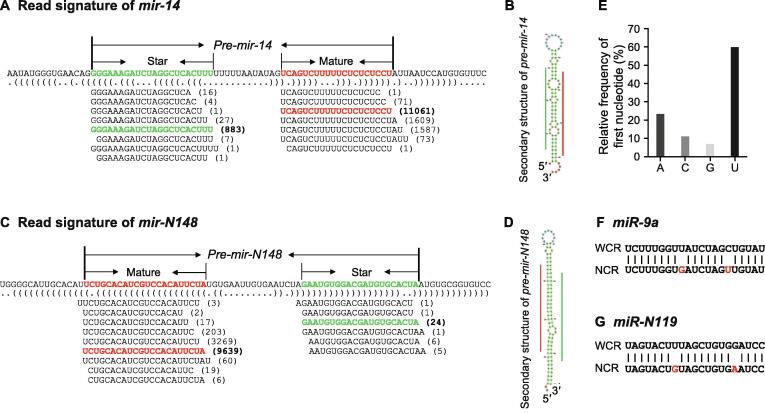
**Identified miRNA candidates in WCR and NCR** **A.** and **C.** Examples of conserved and non-conserved miRNA candidates, *mir*-*14* and *mir*-*N148*, respectively. *mir*-*14* is a conserved miRNA, while *mir*-*N148* is corn rootworm-specific. For each miRNA, listed information includes pri-miRNA excerpt, pre-miRNA, secondary structure in dot-bracket notation, and read abundance along the precursor. Letters with red and green colors indicate mature and star miRNAs, respectively, while numbers within brackets show the copy number of small reads from sRNA-seq dataset. **B.** and **D.** Secondary structures of *pre*-*mir*-*14* and *pre*-*mir*-*N148* in WCR, predicted by RNAfold [26]. Red line indicates the position of the mature miRNA, while green line shows the position of the star miRNA. **E.** Relative frequency of first nucleotide of mature miRNAs in WCR. **F.** and **G.** Examples showing nucleotide changes of mature miRNAs between WCR and NCR. (F) shows a deeply conserved candidate (*miR*-*9a*), while (G) displays a corn rootworm-specific one (*miR*-*N119*). pri-miRNA, primary microRNA; pre-miRNA, precursor miRNA.

In the last decade, the combination deep sequencing of sRNA libraries, whole genome reference sequences, and bioinformatic mining has become a popular and powerful approach to identify miRNAs [25]. With this method many miRNAs were uncovered in diverse species [20], [21]. Using the 18 WCR sRNA samples described above, and an in-house sequenced draft genome of WCR (data not shown), we used the pipeline shown in Figure S3 to identify miRNAs in WCR. In short, after candidates were retrieved, their mature and star miRNAs were aligned to known miRNAs in Arthropoda species in miRBase (version 21) [22] to explore their conservation, which led to a collection of conserved and non-conserved miRNAs. Sequence similarity search was then carried out to identify miRNA candidates in NCR by comparing reads from two NCR sRNA samples and candidates in WCR (Figure S3). This method identifies miRNAs with high confidence since it considers many unique features of miRNAs, and not just their mature miRNA sequences. One of its advantages is the use of the entire pre-miRNAs in conjunction with genomic sequences, which are also examined by secondary structure scan. The second advantage is that reads are aligned in the pre-miRNAs, which informs how mature and star miRNAs are generated from their pre-miRNAs. Both features add much confidence in a candidate pre-miRNA, including a standard stem-loop secondary structure and reads corresponding to both mature and star miRNAs. Thus, via this method, we identified the collection of miRNAs in WCR, with comprehensive information not only by exploiting mature and star miRNA sequences but also by including pre-miRNAs and read signatures along each miRNA. Figure 2A and B showed an example dataset retrieved for *pre*-*mir*-*14*, a well-studied miRNA, including its read distribution and structural information [26]. Additionally, a high-confidence corn rootworm-specific pre-miRNA, *dvi*-*mir*-*N148*, is illustrated in Figure 2C and D.

Following stringent criteria (details in Materials and methods), in WCR we uncovered 101 conserved pre-miRNAs, which have mature miRNAs that could be found in other Arthropoda species (Table 1), and 277 non-conserved pre-miRNAs (Table S3). In addition, over 80% mature miRNAs from these 378 pre-miRNAs start with A or U (Figure 2E), consistent with previous observations that mature miRNAs primarily have A or U at their 5′-ends [27]. The 101 conserved pre-miRNAs belong to 67 miRNA families, having multiple miRNA copies or members (Table 1). On the other hand, the 277 non-conserved pre-miRNAs can be grouped into 196 families (Table S3). Among the 101 conserved pre-miRNAs uncovered in this study, 88 have mature miRNA counterparts (or both mature and star miRNA counterparts) in our NCR sRNA libraries (Table 1). However, only 103 of the 277 non-conserved pre-miRNAs had mature miRNA counterparts in our NCR sRNA libraries (Table S3). Interestingly, among these WCR and NCR counterpart miRNAs, 40 miRNAs (Figure 2F and G; Table 1, Table S3) showed single nucleotide polymorphisms (SNPs), indicating that changes in miRNAs exist despite WCR and NCR being closely phylogenetically related.

**Table 1:** **Conserved miRNAs in WCR and NCR**

**List**	**Family**	**Member**	**Mature miRNA (5′→3′)**	**NCR**
1	*bantam*	*dvi-bantam*	UGAGAUCAUUGUGAAAGCUGUUU	√
2	*let-7*	*dvi-let-7*	UGAGGUAGUAGGUUGUAUAGU	√
3	*mir-iab-4*	*dvi-mir-iab-4*	ACGUAUACUGAAUGUAUCCUGA	√
4	*mir-1*	*dvi-mir-1*	UGGAAUGUAAAGAAGUAUGGA	√
5	*mir-2*	*dvi-mir-2a* *dvi-mir-2b* *dvi-mir-2c*	UCACAGCCAGCUUUGAUGAG UCACAGCCAGCUUUGAUGAG UCACAGCCAGCUUUGAUGAG	√ √ √
6	*mir-7*	*dvi-mir-7*	UGGAAGACUAGUGAUUUUGUUGUU	√
7	*mir-8*	*dvi-mir-8*	UAAUACUGUCAGGUAAAGAUGUC	√
8	*mir-9*	*dvi-mir-9a* *dvi-mir-9b* *dvi-mir-9c* *dvi-mir-9d* *dvi-mir-9e*	UCUUUGGUGAUCUAGUUGUAUG UCUUUGGUUAUCUAGCUGUAUGA UCUUUGGUGAUCUAGUUGUAUG UCUUUGGUGAUCUAGUUGUAUG UAGUACUUUAGCUGUAGAUCC	√ √ √ √ √
9	*mir-10*	*dvi-mir-10*	UACCCUGUAGAUCCGAAUUUGU	√
10	*mir-11*	*dvi-mir-11*	CAUCACAGGCAGAGUUCUAGCU	√
11	*mir-12*	*dvi-mir-12*	GGAGUAUUACAUCAGGUACUGGU	√
12	*mir-13*	*dvi-mir-13a* *dvi-mir-13b*	UAUCACAGCCACUUUGAUGAGCU UAUCACAGCCAUUUUGACGAGU	√ √
13	*mir-14*	*dvi-mir-14*	UCAGUCUUUUUCUCUCUCCUAU	√
14	*mir-29*	*dvi-mir-29*	UAGCACCAUUCGAAAUCAGUUC	√
15	*mir-34*	*dvi-mir-34*	UGGCAGUGUGGUUAGCUGGUUGUG	√
16	*mir-71*	*dvi-mir-71*	UCUCACUACCUUGUCUUUCAUG	√
17	*mir-87*	*dvi-mir-87*	GUGAGCAAAGAUUCAGGUGUAU	√
18	*mir-92*	*dvi-mir-92a* *dvi-mir-92b* *dvi-mir-92c*	UAUUGCACUAGUCCCGGCCUAU AAUUGCACUUGUCCCGGCCUGC UAUUGCACCAGUCCCGGCCUGA	√ √ √
19	*mir-100*	*dvi-mir-100*	AACCCGUAGAUCCGAACUUGUG	√
20	*mir-124*	*dvi-mir-124a* *dvi-mir-124b* *dvi-mir-124c*	UAAGGCACGCGGUGAAUGCCA UAAGGCACGCGGUGAAUGCCA UAAGGCACGCGGUGAAUGCCA	√ √ √
21	*mir-125*	*dvi-mir-125*	UCCCUGAGACCCUUACUUGUGA	√
22	*mir-133*	*dvi-mir-133a* *dvi-mir-133b*	UUGGUCCCCUUCAACCAGCUGU UUGGUCCCCUUCAACCAGCUGU	√ √
23	*mir-137*	*dvi-mir-137*	UAUUGCUUGAGAAUACACGUAG	√
24	*mir-184*	*dvi-mir-184*	UGGACGGAGAACUGAUAAGGGC	√
25	*mir-190*	*dvi-mir-190*	AGAUAUGUUUGAUAUUCUUGGUUG	√
26	*mir-193*	*dvi-mir-193*	UACUGGCCUGUUAAGUCCCAAG	√
27	*mir-210*	*dvi-mir-210*	CUUGUGCGUGUGACAGCGGCU	√
28	*mir-219*	*dvi-mir-219*	UGAUUGUCCAAACGCAAUUC	√
29	*mir-252*	*dvi-mir-252*	CUAAGUACUAGUGCCGCAGGAG	√
30	*mir-263*	*dvi-mir-263a* *dvi-mir-263b*	AAUGGCACUAGAAGAAUUCACG CUUGGCACUGGAAGAAUUCACAGA	√ √
31	*mir-275*	*dvi-mir-275a* *dvi-mir-275b*	UCAGGUACCUGAAGUAGCGCG UCAGGUACCUGAAGUAGCGCG	√ √
32	*mir-276*	*dvi-mir-276*	UAGGAACUUCAUACCGUGCUCU	√
33	*mir-277*	*dvi-mir-277a* *dvi-mir-277b*	UAAAUGCACUAUCUGGUACGACA UAAAUGCACUAUCUGGUACGACA	√ √
34	*mir-279*	*dvi-mir-279a* *dvi-mir-279b*	UGACUAGAUCCACACUCAUUAA UGACUAGAUGGAACACUCGCCU	√ √
35	*mir-281*	*dvi-mir-281a* *dvi-mir-281b*	AAGAGAGCUAUCCGUCGACAGU AAGAGAGCUAUCCGUCGACAGU	√ √
36	*mir-282*	*dvi-mir-282*	UAGCCUCUCCUAGGCUUUGUCU	√
37	*mir-283*	*dvi-mir-283*	AAAUAUCAGCUGGUAAUUCUGGG	√
38	*mir-305*	*dvi-mir-305a* *dvi-mir-305b*	AUUGUACUUCAUCAGGUGCUC AUUGUACUUCAUCAGGUGCUC	√ √
39	*mir-307*	*dvi-mir-307a* *dvi-mir-307b*	UCACAACCUCCUUGAGUGAGC UCACAACCUCCUUGAGUGAGC	√ √
40	*mir-315*	*dvi-mir-315*	UUUUGAUUGUUGCUCAGAAAGCC	√
41	*mir-317*	*dvi-mir-317a* *dvi-mir-317b*	UGAACACAGCUGGUGGUAUCUCAGU UGAACACAGCUGGUGGUAUCUCAGU	√ √
42	*mir-750*	*dvi-mir-750a* *dvi-mir-750b*	CCAGAUCUAACUCUUCCAUGCGA CCAGAUCUAACUCUUCCAUGCGA	√ √
43	*mir-927*	*dvi-mir-927* *dvi-mir-927b*	UUUAGAAUUCCUACGCUUUACC UUUAGAAUCUGUACGCUUUGUU	√ √
44	*mir-929*	*dvi-mir-929*	AAAUUGACUCUAGUAGGGAGU	√
45	*mir-932*	*dvi-mir-932*	UCAAUUCCGUAGUGCAUUGCAGU	√
46	*mir-965*	*dvi-mir-965*	UAAGCGUAUAGCUUUUCCCCU	√
47	*mir-970*	*dvi-mir-970*	UCAUAAGACACACGCGGCUGU	√
48	*mir-971*	*dvi-mir-971*	CACUCUAAGUUUGAACACCAAGC	√
49	*mir-980*	*dvi-mir-980*	UAGCUGCCUUUUGAAGGGCAAU	√
50	*mir-981*	*dvi-mir-981*	UUCGUUGUCGACGAAACCUGCA	√
51	*mir-989*	*dvi-mir-989*	UGUGAUGUGACGUAGUGGUAUG	×
52	*mir-993*	*dvi-mir-993*	GAAGCUCGUCUCUACAGGUAUCU	√
53	*mir-995*	*dvi-mir-995*	UAGCACCACAUGAUUCAGCUUA	√
54	*mir-998*	*dvi-mir-998*	UAGCACCAUGGGAUUCAGCUC	√
55	*mir-1000*	*dvi-mir-1000*	AUAUUGUCCUGUCACAGCAGU	√
56	*mir-1001*	*dvi-mir-1001a* *dvi-mir-1001b*	ACAGCUUUAAAAUCCCAAGGAUCCU ACAGCUUUAAAAUCCCAAGGAUCCU	× ×
57	*mir-1175*	*dvi-mir-1175*	UGAGAUUCAACUCCUCCAACUUAG	√
58	*mir-2514*	*dvi-mir-2514a* *dvi-mir-2514b* *dvi-mir-2514c* *dvi-mir-2514d*	AUUCAUUACAGUGGUCCUCUGUGCU AUUCAUUACAGUGGUCCUCUGUGCU AUUCAUUACAGUGGUCCUCUGUGCU AUUCAUUACAGUGGUCCUCUGUGCU	× × × ×
59	*mir-2765*	*dvi-mir-2765*	UGGUAACUCCACCACCGUUGGCG	√
60	*mir-2779*	*dvi-mir-2779a* *dvi-mir-2779b* *dvi-mir-2779c* *dvi-mir-2779d* *dvi-mir-2779e* *dvi-mir-2779f*	AUCCGGCUCGAAGGACCA AUCCGGCUCGAAGGACCA AUCCGGCUCGAAGGACCA AUCCGGCUCGAAGGACCA AUCCGGCUCGAAGGACCA AUCCGGCUCGAAGGACCA	× × × × × ×
61	*mir-2788*	*dvi-mir-2788*	CAAUGCCCUUGGAAAUCCCAA	√
62	*mir-2796*	*dvi-mir-2796*	GUAGGCCGGCGGAAACUACUUGC	√
63	*mir-2944*	*dvi-mir-2944a* *dvi-mir-2944b* *dvi-mir-2944c*	UAUCACAGCCAGUAGUUACCU UAUCACAGCCAGUAGUUACCU UAUCACAGCCAGUAGUUACCU	√ √ √
64	*mir-3049*	*dvi-mir-3049*	UCGGGAAGACAGUUGCGGCGGAUU	√
65	*mir-3477*	*dvi-mir-3477a* *dvi-mir-3477b*	UAAUCUCAUUUGGUAACUGUGA UAAUCUCAUUUGGUAACUGUGA	√ √
66	*mir-3849*	*dvi-mir-3849*	ACAUUUUAACCAUAGUGCUGUU	√
67	*mir-6012*	*dvi-mir-6012*	UUCGGCGAUAAGAUCAGCCUGU	√

*Note*: ‘√’ indicates that the mature miRNA identified in WCR was also detected in NCR; ‘×’ indicates that the mature miRNA identified in WCR was not detected in NCR. miRNA, microRNA; WCR, western corn rootworm; NCR, northern corn rootworm.

### Conservation and divergence of WCR miRNAs

**Figure 3:**
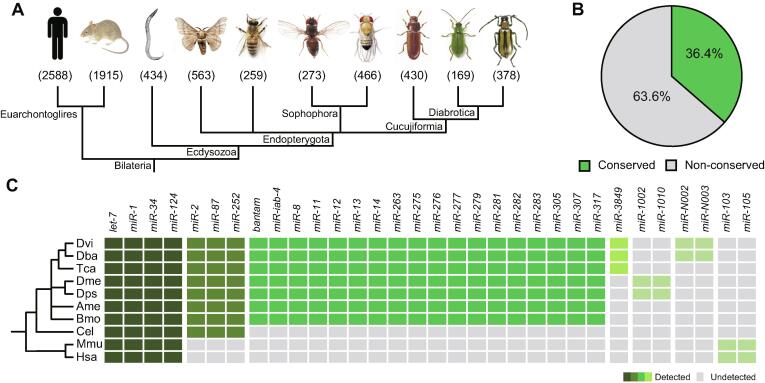
**Conservation of WCR miRNAs across Bilateria** **A.** Phylogenetic representation of ten selected species for conservation analysis. Number below the species picture indicates the number of mature miRNAs available in public database (miRBase, version 21) or identified in this study. **B.** Percentage of conserved and non-conserved miRNAs in ten selected species. **C.** Phylogenetic distribution of conserved miRNA families. The matrix consists of ten selected species and 32 representative miRNA families (columns). If a miRNA or miRNA family was annotated in miRBase and detected in this study, the box is highlighted in different shades of green. Otherwise, the box is covered by gray. Hsa, *homo sapiens* (human); Mmu, *Mus musculus* (house mouse); Cel, *Caenorhabditis elegans*; Bmo, *Bombyx mori* (silkworm); Ame, *Apis mellifera* (western honey bee); Dps, *Drosophila pseudoobscura*; Dme, *D*. *melanogaster* (fruit fly); Tca, *Tribolium castaneum* (red flour beetle); Dba, *Diabrotica barberi* (northern corn rootworm); Dvi, *D*. *virgifera virgifera* (western corn rootworm).

Similarly to protein-coding genes, previous research uncovered that miRNAs have different degrees of conservation [28], [29]. In the process of miRNA identification (Figure S3), we combined and exploited all known miRNAs in Arthropoda and found that 101 pre-miRNAs in WCR belong to 67 families (Table 1). To further study the conservation of these WCR miRNAs across Bilateria kingdoms, we queried additional model animal species including human (*homo sapiens*), house mouse (*Mus musculus*), *C*. *elegans*, and model species in phylum Arthropoda*,* including silkworm (*Bombyx mori*), western honey bee (*Apis mellifera*), *Drosophila pseudoobscura*, fruit fly (*D. melanogaster*), and red flour beetle (*Tribolium castaneum*) (Figure 3A), all of which have a complete/updated miRNA datasets in miRBase (version 21) [22]. In total, 7475 miRNAs were scanned. When a conserved miRNA was defined as one that is present in two or more species, we found that only 36.4% of miRNAs are conserved, while the rest are not among the ten compared species (Figure 3B; Table S4), suggesting that the miRNA pools across species are dynamic and that each species has a relative large pool of species-specific miRNAs which are potentially evolutionarily transient [28], [30].

Multispecies analysis of miRNAs revealed that their conservation level is associated with the phylogenetic relationship among species (Figure 3C). Four miRNA families (*let*-*7*, *miR*-*1*, *miR*-*34*, and *miR*-*124*) are highly conserved in all ten selected species, pointing to the important processes they regulate in Bilateria. There are three families, *miR*-*2*, *miR*-*87*, and *miR*-*252*, only appearing on Ecdysozoa but not in human or rat (Figure 3C). A significant number of miRNA families (more than 20) are specific to insects (Figure 3C; Table S4). Their evolution is potentially related to specialized gene regulatory functions evolved in insects. For instance, *miR*-*8* is homologous to *miR*-*200*, but is only detected in insects, and is believed to play a role in insect larval nervous system development [28], [29]. We also found that some miRNAs are not consistently associated with one phylogenetic branch (Table S4). Taking *miR*-*7*, *miR*-*9*, and *miR*-*10* as examples, these miRNAs exist in human, mouse, and all insect species surveyed in this study; however, they are absent in *C. elegans* (Table S4). It is also possible that the lack of complete genomic information results in many miRNAs that do not appear to be deeply conserved.

Apart from conserved miRNAs, we identified 277 pre-miRNAs that are only detected in WCR and NCR and they are potentially specific to *Diabrotica* (Table S3). Further, 174 pre-miRNAs were only detected in WCR and potentially are WCR specific. Of the above, 100 pre-miRNAs were detected only in 1st and 2nd instar of WCR, when compared to samples from the same life stages of NCR (Table S5). Conceivably, WCR-specific miRNAs might have counterparts in NCR, which were not identified since only 1st and 2nd instar samples from NCR were sequenced. A large number of potential WCR-specific miRNAs supports the presence of a dynamic and rapidly evolving pool of new miRNAs in WCR, as previously observed in other organisms [28], [30].

### Temporal expression patterns of miRNAs in WCR

The collection of rootworm reads that correspond to miRNAs were generated from a sizable population of sRNA libraries, and this number of reads provided quantitative profiling information for miRNAs. It is well established that the normalized read frequencies of miRNAs can be used to quantify the expression level of the miRNAs [21]. The 18 biological WCR samples were prepared from six different life stages, with three replicates per stage (Table S1), which allowed us to systematically examine the pattern of miRNA expression.

**Figure 4:**
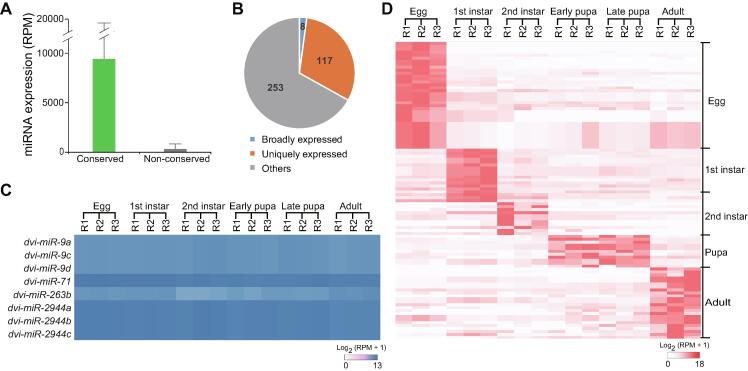
**Diverse expression of miRNAs in WCR** **A.** Expression values of conserved and non-conserved miRNAs. Data are shown as mean ± SD. **B.** Distribution of miRNAs based on their expression in WCR developmental stages. Broadly expressed miRNAs are highly expressed in all life stages (RPM > 100), while uniquely expressed miRNAs are only highly expressed in one of the six analyzed life stages. “Others” are miRNAs that are not grouped into broadly and uniquely expressed categories. **C.** Heatmap showing the expression patterns of eight broadly expressed miRNAs. **D.** Heatmap showing the expression patterns of uniquely expressed miRNAs. All of expression values of miRNAs in (A, C, and D) are calculated with data in Table S6 (details in Materials and methods). RPM, reads per million.

As expected, we found that miRNA expression levels varied greatly among different miRNAs, and that the expression of many specific miRNAs changed dramatically during development. When we examined conserved and non-conserved miRNAs, we found that the expression of conserved miRNAs is, in general, much higher than that of non-conserved miRNAs (Figure 4A), most likely because non-conserved miRNAs may merge into regulatory mechanisms later in time and are subject to lower selective constraint [31]. In fact, the expression pattern of conserved miRNAs tended to be similar among species when we compared the expression patterns of their counterparts in fruit fly [32]. For instance, *miR*-*100* and *let*-*7* are miRNAs that are abundant in pupal and adult stages in WCR and fruit fly (Figure S4), potentially having similar regulatory roles during these life stages in both insects.

Based on the variation of expression in different life stages, we divided miRNAs into three categories: 1) broadly expressed (stably expressed at a consistent level in six life stages), 2) uniquely expressed (expression in one life stage significantly higher than in other life stages), and 3) others [expression pattern not belonging to categories 1) or 2)]. We found that only a small fraction of miRNAs (8 miRNAs, around 2% of the total miRNAs) (Figure 4B and C; Table S6) are broadly expressed. Approximately one third of all miRNAs are highly expressed in only one life stage, ranging from egg to adult, indicating that these miRNAs are life stage-specific (Figure 4D; Table S6). Taken together, these results indicate that miRNA expression in the explored WCR life stages is extremely variable, and suggest that miRNAs play fundamental and specific regulatory roles throughout rootworm development.

### Dynamic changes of miRNA clusters

In well-studied organisms, such as humans [33] and fruit fly [34], many miRNAs are known to co-localize to miRNA clusters. These miRNAs are co-expressed and co-regulated (polycistronic miRNAs), and are expected to jointly regulate molecular pathways either by co-targeting individual genes or by targeting different components of the same pathway [35]. The availability of a draft genome in combination with a detailed collection of miRNAs and their expressing levels in WCR, enabled us to scan clustered miRNAs, and compare the expression data across the members of the same miRNA cluster. Following an exhaustive search (details in Materials and methods), we identified 20 miRNA clusters (Table S7). Several clusters contain conserved miRNAs, while others contain non-conserved miRNAs. Among the conserved miRNA clusters, a few of them have been well studied in other species, such as the *mir*-*2*/*mir*-*13* cluster [36] and the *let*-*7*/*mir*-*100* cluster [37]. Several clusters identified in previous studies were also detected in our research, indicating that these clusters are evolutionarily conserved.

**Figure 5:**
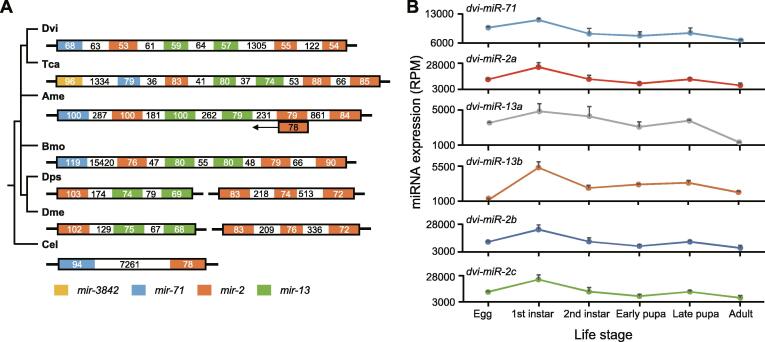
**Evolution ofthe*mir*-*2* and *mir*-*13* cluster and its expression in WCR** **A.** Diagram showing the *mir*-*2*/*mir*-*13* cluster in each selected model species. Numbers shaded by colors indicate the length of each pre-miRNA, while numbers with white background are distances between these pre-miRNAs. Location and length information is from miRBase (version 21), except that the distance information in WCR is from our draft genome. **B.** The expression pattern of miRNAs in the *mir*-*2*/*mir*-*13* cluster in WCR. Expression pattern was drawn based on the expression values in Table S6.

Even though conserved clusters were present in selected model species, we found that notable changes emerged in these clusters among different species. Using the *mir*-*2*/*mir*-*13* cluster as an example, a search was conducted across nine selected model species. As shown in Figure 5A, we did not find counterparts of the *mir*-*2*/*mir*-*13* cluster in human and rat. In nematode (*C. elegans*), a large gap (> 7 kb) was detected within the cluster of *mir*-*2* and *mir*-*71*, while in flies (*D. pseudoobscura* and *D. melanogaster*) there are two clusters of *mir*-*2* and *mir*-*13* located on two chromosomes (Figure 5A). In other four examined Arthropoda species, *mir*-*71* is incorporated into the *mir*-*2*/*mir*-*13* cluster, yielding a new cluster that contains a scaffold consisting of a copy of *mir*-*71*, a copy of *mir*-*2*, two copies of *mir*-*13*, and two copies of *mir*-*2* (Figure 5A). Intriguingly, although this scaffold exists in these four Arthropoda species (Figure 5A), there is much variation between species. For instance, in silkworm, a large intergenic gap (> 15 kb) appears between *mir*-*71* and the first copy of *mir*-*2*; in red flour beetle, *mir*-*3842*, a relatively new miRNA, appears incorporated into the cluster; and there is a *mir*-*2* member located in the complementary strand in honey bee. Albeit the cluster centered on *mir*-*2* and *mir*-*13* exists in these species, our data point to a dynamic evolutionary change that is supported by the cluster differences presented in the examined species. Moreover, another conserved cluster, *let*-*7*/*mir*-*100*, presented high variation among the studied species (Figure S5A).

When we scanned *mir*-*71, mir*-*2,* and *mir*-*13* in WCR, it was apparent that their expression patterns were directly correlated across the six life stages, indicating that they are co-expressed and/or co-regulated (Figure 5B). Similarly, *let*-*7* and *mir*-*100* exhibit patterns of co-expression/regulation (Figure S5B).

### miRNA-guided cleavage as a potential regulatory mechanism in WCR suggested by PARE sequencing

**Figure 6:**
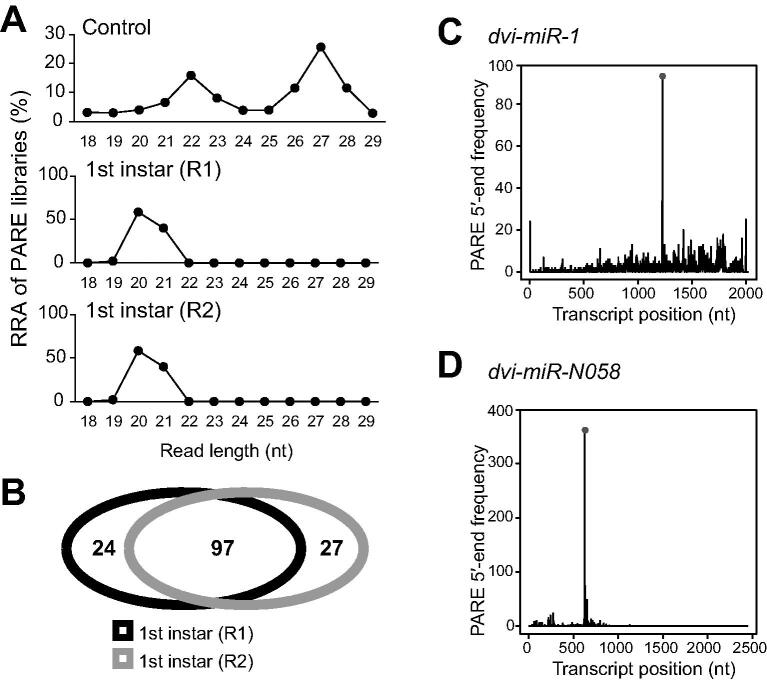
**Identificationof mRNA targets of miRNAsby PARE sequencing** **A.** RRA and read length distribution of PARE libraries. A conventional sRNA library was used as a control. **B.** Venn diagram showing the numbers of mRNA targets of miRNAs identified by two PARE libraries. **C.** Validation of the cleavage ofa gene transcript encoding a GPI-anchored protein by a conserved miRNA, *dvi*-*miR*-*1*. **D.** Validation of the cleavage of a gene transcript encoding a conserved hypothetical protein by a WCR-specific miRNA, *dvi*-*miR*-*N058*. PARE, Parallel Analysis of RNA Ends; GPI, glycosylphosphatidylinositol.

miRNAs in animals generally recognize their target mRNAs at 3′ UTRs through as few as 6–8 complementary nucleotides at the 5′-ends of the miRNAs to inhibit mRNA translation [38], [39]; while in plants, miRNAs usually have near-perfect pairing with their mRNA targets, not limited to 3′ UTRs, and function to guide cleavage of target mRNAs [40]. Whereas the primary function of miRNAs in animals is to bind to their mRNA targets and block protein translation, there are reports suggesting that animal miRNAs also can down-regulate their targets by guiding cleavage of target mRNAs [41]. To understand whether this type of miRNA-guided cleavage exists in WCR, we deeply sequenced two PARE libraries [42] prepared from two samples of 1st instar WCR, and a conventional sRNA library was used as a control (Table S8). As shown in Figure 6A, an expected difference in read length distribution was observed between the PARE libraries and the control. There are two peaks at 22 nt and 27 nt in the conventional sRNA library, and a single peak at 20 nt in the PARE libraries, suggesting that the PARE libraries successfully captured the 5′-ends of degraded RNAs [42]. In order to detect as many cleavage sites as possible, we sequenced each library with a depth of over 130 million reads (Table S8).

To analyze the two PARE libraries, we used an in-house WCR transcriptome harboring 63,732 transcripts (data not shown) and all miRNA candidates to search miRNA cleavage sites in WCR (see details in Materials and methods). Overall, 148 high-confidence miRNA–target pair candidates meeting our criteria were detected (Figure 6B). Specifically, 97 of the aforementioned potential targets were found in both PARE libraries, while samples 1 and 2 had 24 and 27 sample-specific potential mRNA targets, respectively (Figure 6B). In total, 121 transcripts could potentially be cleaved by 81 miRNAs. Since we only sequenced the PARE libraries from a single life stage, 1st instar, it is possible that additional potential mRNA targets exist in WCR that are regulated by miRNA-guided cleavage. Figure 6C showed a conserved miRNA, *dvi*-*miR*-*1*, and its possible cleavage site located in the mRNA target encoding glycosylphosphatidylinositol (GPI)-anchored protein, and Figure 6D illustrated a WCR-specific miRNA, *dvi*-*miR*-*N058*, and its potential mRNA target (encoding a conserved hypothetical protein). Both miRNA-directed potential cleavage sites are located in coding regions, indicating that a miRNA cleavage site may not be limited to the 3′ UTRs of target transcripts.

## Discussion

WCR is highly adaptable to environmental challenges and able to quickly become resistant to insecticides and insecticidal proteins and gain phenotypes such as behavioral changes in response to agricultural practices [43]. The genetic and molecular bases for these phenotypic changes as well as WCR developmental biology are largely unknown. Here, we sequenced sRNA pools and characterized piRNAs and miRNAs across life stages of WCR. The sRNA information gained from NCR further strengthened the confidence of newly identified rootworm-specific miRNAs. This information not only reveals the complexity of sRNA transcriptome, showing dynamic and evolutionary changes, but also provides a resource for investigating biological questions relevant to basic WCR biology and pest control.

Using next generation sequencing, we uncovered an abundance of total and unique reads of piRNAs and miRNAs, the two largest classes of non-coding RNA molecules [13], [14], and demonstrated that in WCR, like in other organisms, piRNAs are the most abundant class of small non-coding RNAs (Figure 1, Figure S2) [13], [14]. Furthermore, we observed that the expression of miRNAs and piRNAs varies throughout life stages, which likely reflects their roles in regulating gene expression during early development (Figure 1C). Specifically, piRNA complexes are mainly linked to epigenetic and post-transcriptional gene silencing of retrotransposons, a process which takes place during critical developmental stages that are linked to cell differentiation [44]. This may explain the need for a higher expression of piRNAs in WCR embryos and a decreasing gradient of piRNA expression from the egg to pupal stages. In the adult stage, the levels of cell differentiation are expected to plateau, tracking with the reduction of piRNA expression. Meanwhile, adult insects engage in complex behaviors and have to respond quickly to environmental stimuli, and these behaviors are likely mediated by temporal changes in gene expression, perhaps facilitated by miRNA shifts [45]. Overall, this dynamic change of sRNA populations across life stages may be related to the regulatory functions of miRNAs and piRNAs.

The present study characterizes WCR miRNAs via comparative analysis of genome sequences and sRNAs using conservative empirical criteria of known miRNAs. Beyond focusing only on mature miRNAs, we interrogated a set of miRNA elements including star miRNAs, pre-miRNAs, and the secondary structures of pre-miRNAs (Figure 2). The aforementioned analyses have increased the confidence of the candidate miRNAs reported here. Capitalizing on miRNAs from well-characterized arthropods and several other organisms in miRBase (version 21) [22], the identified 378 pre-miRNAs were classified into 101 conserved and 277 corn rootworm-specific pre-miRNAs (Table 1, Table S3). The high abundance of WCR-specific miRNAs and the diversity of miRNAs between WCR and NCR strongly indicate that miRNAs in these closely-related species are undergoing a transient selection. This observation is quite similar to the discoveries in closely-related plants, *Arabidopsis thaliana* and *A. Lyrata* [46], which suggests that many species-specific miRNAs move rapidly in and out of miRNA pools. This hypothesis was further strengthened when we found that most WCR miRNAs exhibit temporal changes in expression. A careful examination of miRNA expression in six different WCR life stages revealed that only 2% of miRNAs (8 miRNAs) in WCR were broadly and consistently expressed (Figure 4; Table S6). In contrast, over 30% of the miRNAs were found to have stage-specific expression (Figure 4B). This is expected when considering that miRNAs generally down-regulate their target genes in a temporal manner [30]. Meanwhile, compared to conserved miRNAs, most of corn rootworm-specific miRNAs are expressed at much lower levels (Figure 4A). It is possible that many of the newly characterized non-conserved miRNAs are not fully functional in miRNA target circuits.

Combining expression values with miRNA clusters, the hypothesis that miRNAs in the same cluster are co-expressed and/or co-regulated and regulate molecular pathways either by co-targeting individual genes or by targeting different components of the same pathway appears well supported by the example cluster of *mir*-*2*, *mir*-*13, and mir*-*71* (Figure 5). The *mir*-*2*/*mir*-*13*/*mir*-*71* cluster has been previously identified in Protostomes and is believed to be absent in Deuterostomes [47], suggesting a specific role in Protostomes. *mir*-*71* is also absent in Vertebrata and Urochordata [47]. Consistent with a unique role in Deuterostomes, including insects, the *mir*-*2* family is known to function in the regulation of the earliest insect developmental genes such as *bicoid* (*bcd*)*,* an early anterior–posterior patterning gene (exemplified in *Drosophila*) [48], *abnormal wing disc* (*awd*) and *fringe* (*fng*), regulators wing formation via Notch signaling (exemplified in silk moth, *B. mori*) [49], and *Krüppel homolog 1* (*Kr-h1*), a juvenile hormone-dependent transcription factor that inhibits metamorphosis in insects (exemplified in German cockroach, *Blattella germanica*) [50]. *mir*-*2*/*mir*-*71* has been shown to respond to viral infection by inducing autophagy in shrimp [51]. *mir*-*2* family is also involved in cell death pathways in insects, regulating insect-specific proapoptotic genes *reaper*, *grim*, and *sickle* [52], [53]. An additional arthropod-specific role for *mir*-*71* is the regulation of chitin synthase [54]. The presence of *mir*-*71*, *mi*r-*2*, and *mir*-*13,* with multiple representatives of *mir*-*2* and *mir*-*13,* represent a robust arthropod miRNA-regulated developmental pathway in WCR. It is also believed that the duplication of miRNAs such as *mir*-*2* and *mir*-*13* may represent an evolutionary acquisition of novel miRNA functions [36]. While the links between miRNAs such as the *mir*-*2*/*mir*-*13*/*mir*-*71* cluster and insecticide resistance are not fully understood, insect-specific miRNA-regulated developmental pathways may provide the plasticity for adaptation to environmental stimuli. Building on the above examples, the association between conservation, expression, and function of miRNAs can be a powerful tool for understanding their functions in diversification and adaptation of insects.

The involvement of miRNAs in insecticide resistance has only recently been uncovered. Studies in common house mosquito, *Culex pipiens*, resistant to pyrethroid insecticides, identified *mir*-*71* along with several other miRNAs as being significantly upregulated in deltamethrin-resistant (DR) mosquito strain [55]. More recently, Guo et al. [10] have confirmed down regulation of all three miRNAs: *miR*-*2*, *miR*-*13*, and *miR*-*71* in *C. pipiens* DR strain and their effects on cytochrome P450 transcripts. Additionally, both *miR*-*278*-*3P* and *miR*-*285* of *C. pipiens* were identified as components of deltamethrin resistance, through regulation of the cytochrome P450 genes [56]. *mir*-*7a* and *mir*-*8519* were also implicated in chlorantraniliprole (diamide) resistance in diamondback moth, *Plutella xylostella,* through their regulation of ryanodine receptor (RyR) [57]. Interestingly, there is also evidence for differential miRNA expression in Bt protein (Cry1Ab)-resistant Asian corn borer (ACB), *Ostrinia furnacalis* [58], pointing to miRNAs being either causal agents or the effectors of both chemical insecticide or insecticidal protein resistance. Our study collected a relative complete list of miRNAs in WCR, and traced their expression patterns and evolutionary tracks, which provides a rich resource for pest science research.

PARE libraries with a high sequencing depth uncovered that guided cleavage of targets by miRNAs potentially exists in WCR, even though it is considered a minor miRNA regulatory mechanism in animals [30], [59]. Still, examples of miRNA-guided cleavage in animals exist [60]. By sequencing PARE libraries in 1st instar WCR, we identified 148 high-confidence miRNA–target pairs with a potential cleavage regulatory mechanism (Figure 6B). Further, the cleavage positions of potential mRNA targets, as identified by PARE, are not limited to 3′ UTR. These observations suggest that in WCR miRNAs could guide cleavage of their target mRNAs along the entire transcripts.

## Conclusion

In conclusion, the sRNA transcriptome in WCR is very dynamic, and as the largest classes of sRNAs, piRNAs and miRNAs change their relative abundance during the insect life circle. This expression gradient is potentially related to the differences in the gene regulatory roles of piRNAs and miRNAs. Many new and species-specific miRNAs exist in WCR. Moreover, differences exist between WCR and NCR, which suggests that the pool of transcribed miRNAs is rapidly selected. Within conserved miRNAs, nucleotide mutations occur continuously. Most miRNAs undergo temporal changes in expression as evidenced by differential expression through the lifecycle of WCR, revealing the complexity of both regulation of miRNAs and regulation via miRNAs. miRNA clusters further uncovered the continuous changes and complex evolutionary dependence. Some conserved clusters, such as *mir*-*71*/*mir*-*2*/*mir*-*13*, undergo changes in miRNA location and miRNA member gains and losses. Observations from PARE data suggest that a significant portion of WCR miRNAs could regulate their target transcripts by a cleavage-guided mechanism. Not only do these systematic analyses reveal the dynamics and complexity of the sRNA transcriptome in corn rootworm, but also provide new insights into the functions and evolution of miRNAs. This critical and rich resource can help facilitate further sRNA research and applications in corn rootworm and other coleopteran insects.

## Materials and methods

### Sample collection

NCR eggs were obtained from the USDA-ARS North Central Agricultural Research Lab, Brookings, SD. The WCR eggs and pupae were purchased from Crop Characteristics (Farmington, MN). Rootworm adults and pupae were flash-frozen and stored at −80 °C prior to RNA extraction, while eggs were further treated and incubated to generate embryos of various developmental stages and larvae. The rootworm eggs were stored in soil at 6 °C. The WCR eggs were incubated for three, five, seven, and ten days at 28 °C, 60% relative humidity (RH) to reach different levels of embryonic development. The eggs were washed from soil with deionized water and sterilized with 10% formaldehyde for 10 min, followed by five washes with water. To produce 1st and 2nd instar larvae, the eggs were placed on filter paper to hatch at 28 °C, 60% RH on petri dishes containing artificial diet. Eggs and neonate larvae were frozen in microcentrifuge tubes on dry ice, while additional larvae were further incubated with artificial diet to reach 2nd instar stage.

RNA samples for sRNA sequencing were extracted from NCR 1st and 2nd instar larvae; WCR eggs, 1st instar larvae, 2nd instar larvae, early pupae, late pupae, and adults. Total RNA was extracted from three replicate samples for each of the life stages. For each sample, approximately 100 μl volume of insect sample was extracted with TRIzol Reagent (Invitrogen, ThermoFisher Scientific, Waltham, MA). The samples were homogenized at room temperature in a 1.5 ml microcentrifuge tube with 200 μl of TRIzol using a pellet pestle (Fisherbrand, Grand Island, NY) and Pestle Motor Mixer (Cole-Parmer, Vernon Hills, IL). Following homogenization, an additional 800 μl of TRIzol was added, the homogenate was vortexed and centrifuged to remove debris. The extraction was then carried out following manufacturer’s protocol. The samples were resuspended in TE and quantified on NanoDrop 8000 (ThermoFisher Scientific). Total RNA from three-, five-, seven-, and ten-day-matured eggs was combined in 1:1:1:1 amount. Total RNA from approximately 600 μl volume of neonate (1st instar) WCR was used as the starting material for the PARE library, which produced ~ 1 mg total RNA.

### sRNA library construction and sequencing

sRNA libraries were prepared using Illumina’s TruSeq small RNA sample preparation kit (Illumina, San Diego, CA) according to the manufacturer’s recommendations. Briefly, RNA 3′ and RNA 5′ adapters were sequentially ligated unto 1 μg of high-quality purified sRNA sample using truncated T4 RNA Ligase 2 and T4 RNA Ligase, respectively. The sRNA fragments were then reverse-transcribed to generate first strand cDNA using SuperScript II reverse transcriptase (Catalog No. 18064014, ThermoFisher Scientific). The cDNA was converted into double-stranded cDNA with PCR using two primers that respectively anneal to the ends of 3′ and 5′ adapters. This process selectively enriches those fragments that have adapter molecules on both ends. The amplified cDNA was then purified on a 6% PAGE gel, normalized to 2 nM concentration, denatured with sodium hydroxide, and diluted in HT1 hybridization buffer (Catalog No. FC-404-2005, Illumina) for loading onto a NextSeq 500 flow cell. Sequencing reactions were carried out according to Illumina’s recommended protocol.

### PARE library construction and sequencing

PARE libraries were constructed following the protocol published by German and his colleagues [42]. Polyadenylated (polyA) RNA was purified from 75 μg of total RNA using Dynabeads mRNA purification kit (Catalog No. 61006, ThermoFisher Scientific). 5′-PARE RNA adapters were ligated onto the 5′-end of the polyA RNA using T4 RNA ligase (Catalog No. AM2141, ThermoFisher Scientific) and cleaned with Dynabeads mRNA purification beads for a second time to remove unligated 5′ adapters. Adapter-ligated RNA was reverse-transcribed using SuperScript III reverse transcriptase (Catalog No. 18080093, ThermoFisher Scientific), using an oligo(dT) primer fused with 3′-adapter sequence to produce first strand cDNA. Second strand cDNA synthesis and amplification by PCR were carried out in a single reaction [98 °C for 60 s, 7 cycles of (98 °C for 30 s, 58 °C for 30 s, 72 °C for 5 min), 72 °C for 7 min, hold at 4 °C]. This was followed by an AMPure XP (Catalog No. A63880, Beckman Coulter, Brea, CA) bead clean up. Double-stranded cDNA molecules were cleaved 20 bp downstream of the 3′-adapter with *Mme1* type II restriction endonuclease (Catalog No. R0637S, New England Biolabs, Ipswich, MA) and ligated onto a double-stranded 3′-DNA adapter. Ligated fragments were purified on a 12% PAGE gel to isolate ~ 63-nt fragments comprising a 22-bp 5′ adapter, a 20-bp *Mme*I-digested tag, and a 21-bp 3′ dsDNA adapter. These PARE library fragments were then PCR amplified [98 °C for 30 s, 15 cycles of (98 °C for 10 s, 58 °C for 30 s, 72 °C for 20 s), 72 °C for 10 min, held at 4 °C] using indexed TruSeq PCR primers and purified a second time with 6% PAGE. PARE libraries were assessed for quality on an Agilent Bioanalyzer High Sensitivity DNA chip (Catalog No. 5067-4626 Agilent Technologies, Santa Clara, CA) and normalized to 2 nM concentration prior to pooling. Pooled libraries were denatured with sodium hydroxide, diluted in HT1 hybridization buffer and sequenced according to Illumina’s recommended protocol.

### miRNA identification in WCR and NCR

A computational pipeline centered on an updated version of miRDP [25] was employed to identify miRNA candidates from deep sequencing data (Figure S3). Raw data from each sequenced library were filtered to keep only reads in the range of 18 to 25 nt. Identical reads were collapsed into FASTA format and used as input for processing by the miRDP core algorithm, which extracts the sequences flanking each anchored read for predicting RNA secondary structure and quantifying the compatibility of the distribution of sRNA reads with Drosha- and Dicer-mediated processing. After progressively processing all mapped reads, candidate miRNAs were scored based on a probabilistic model [25].

The following specific settings and modifications were employed in this study: 1) 150 nt was used as the length for extracting reference genome flanking mapped reads; 2) 56 nt was set as the minimum length to filter identified pre-miRNAs; 3) a BLAST search was conducted to filter putative pre-miRNAs that match known plant tRNAs [61] and rRNAs [62] with the cutoffs set as length ≥ 80% and identity ≥ 90%; and 4) the minimal number of reads corresponding to the mature miRNAs was set at 10 reads per million (RPM) as a filtering criterion.

After compiling a miRNA dataset by consolidating retrieved miRNAs and pre-miRNAs from individual libraries, a similarity search was carried out against all Arthropoda mature miRNAs in miRBase (version 21) [22] with two mismatches allowed using the mature miRNAs as queries. By this search, newly found miRNAs that are conserved in other species were identified. To achieve higher confidence when identifying novel non-conserved miRNAs, we also considered reads from sRNA libraries corresponding to miRNA.

### miRNA conservation analysis

Within miRBase (release 21) [22], model species in animal spectrum with relatively complete miRNA collections were selected, including human (*h*. *sapiens*), house mouse (*M. musculus*), *C. elegans*, silkworm (*B. mori*), western honey bee (*A. mellifera*), *D. pseudoobscura*, fruit fly (*D. melanogaster*), and red flour beetle (*T. castaneum*). Mature miRNA candidates in WCR were aligned to the collection of all mature miRNAs in the selected species. Briefly, widely accepted criteria were employed to identify the conserved counterparts between two aligned mature miRNAs [25], including: 1) ≥ 20 bp aligned and 2) mismatches ≤ 2 bp.

### miRNA expression analysis in different life stages in WCR

An initial table including all raw read numbers corresponding to the mature miRNAs in each of the sRNA libraries was compiled. Then, edgeR, a Bioconductor package for differential expression analysis of digital gene expression data [63], was utilized to find differentially expressed miRNAs. Samples from each rootworm life stage were compared to others. Differentially expressed miRNAs were selected when more than two-fold change at *P* < 0.05 were achieved. When comparing samples from early and late pupae, less than five miRNAs were identified to be differentially expressed, thus, samples from early and late pupae were combined together as a pupal group. If one miRNA in one life stage was differentially expressed from the other four life stages, that miRNA was considered a life stage-specific miRNA. In contrast to life stage-specific miRNAs, if a miRNA was not significantly differentially expressed in each life stage, it was categorized as broadly expressed if its mean of RPM mapped value was larger than 50. The remaining miRNAs were classified as “others”.

### miRNA cluster scanning

A combination of the criteria from Altuvia et al. [33] and Chan et al. [64] were used to scan for miRNA clusters in WCR. In detail, miRNAs in one cluster are located on the same scaffold and the same strand, and the gap between the neighboring miRNAs is no more than 3 kb. miRNA clusters of other selected model species were obtained from miRBase (version 21) [22]**.**

### Search mRNA targets of miRNAs by analyzing PARE sequencing data

CleaveLand, a pipeline for using PARE data to find cleaved sRNA targets [65], was employed to search mRNA targets of miRNAs. The sequencing reads from PARE libraries were first mapped to an in-house transcriptome dataset of WCR harboring 63,732 transcripts (data not shown). Then, all mature miRNAs and the aforementioned transcriptome were used to predict miRNA-mRNA target pairs. After complementarity search and signal-to-noise analysis, over 2000 miRNA-mRNA target pair candidates were divided into five categories as described in Addo-Quaye and his colleagues [65]. Only candidates meeting constraint criteria, that is, candidates in categories 0–2 and with *P* value less than 0.05, were finally selected.

## Data availability

The sequencing data used in this study have been deposited in the BioProject database in NCBI (BioProject: PRJNA588643), which are publicly accessible at https://www.ncbi.nlm.nih.gov/, and also in the Genome Sequence Archive [66] in the National Genomics Data Center, Beijing Institute of Genomics, Chinese Academy of Sciences / China National Center for Bioinformation (GSA: CRA003835 with BioProject: PRJCA004377), which are publicly accessible at https://ngdc.cncb.ac.cn.

## CRediT author statement

**Xiaozeng Yang:** Conceptualization, Methodology, Data curation, Writing - original draft, Writing - review & editing, Visualization. **Elane Fishilevich:** Resources, Validation. **Marcelo A. German:** Conceptualization, Writing - review & editing. **Premchand Gandra:** Resources, Data curation. **Robert E. McEwan:** Methodology. **André Billion:** Data curation, Validation. **Eileen Knorr:** Validation. **Andreas Vilcinskas:** Supervision. **Kenneth E. Narva:** Conceptualization, Writing - review & editing, Supervision. All authors have read and approved the final manuscript.

## Competing interests

Kenneth E. Narva, Marcelo A. German, Premchand Gandra, and Robert E. McEwan are current employees of Dow AgroSciences LLC. Xiaozeng Yang and Elane Fishilevich are former employees of Dow AgroSciences LLC. All authors state that they adhere to Elsevier’s Ethics polices.

## Supplementary material

Supplementary Figure S1 **PCA analysis of small RNA libraries from six life stages of WCR**. Multidimensional scaling (MDS) analysis was performed based on the matrix of Euclidean distances and visualized using the first two components k1 and k2 with R v.3.5.1. The first dimension (73%) and second dimension (14%) were shown with X and Y axis, respectively. Samples collected from different stages were represented with different colors. PCA, principal component analysis; EG, egg; FI, 1st instar; SI, 2nd instar; EP, early pupa; LP, late pupa; AD, adult. Abbreviations similarly hereinafter.

Supplementary Figure S2 **Summary of unique reads in WCR and NCR small RNA libraries**. **A.** Relative abundance of unique reads and length distribution in six life stages of WCR. For each life stage, there are three biological replicates. **B.** Unique read abundance and length distribution in 1st and 2nd instar NCR. RRA, read relative abundance; WCR, western corn rootworm; NCR, northern corn rootworm; EG, egg; FI, 1st instar; SI, 2nd instar; EP, early pupa; LP, late pupa; AD, adult.

Supplementary Figure S3 **Flowchart of identification of miRNAs in WCR and NCR**. A parallelogram represents input or output while a rectangle displays a process. Sequences of tRNA and rRNA from GtRNAdb-Genomic tRNA database (Chan and Lowe 2009) and Silva-rRNA database (Quast C et al. 2013), respectively, were used to filter reads generated from tRNAs and rRNAs. miRNAs in Arthropoda are from miRBase (version 21) (Kozomara and Griffiths-Jones 2014).

Supplementary Figure S4 **Similarities of expression patterns of conserved miRNAs in WCR and fruit fly**. **A**. Northern blots in fruit fly are adapted from (Sempere LF et al. 2003). Life stages in fruit fly are embryos (E), larvae (L1-L3), pre-pupae (pp), pupae (pupa), and adult females (♀), respectively. **B**. Expression values in WCR are from Supplementary Table S6. Values in pupae are a combination of early and late pupal stages. WCR, western corn rootworm; EG, egg; FI, 1st instar; SI, 2nd instar; PU, pupa; AD, adult.

Supplementary Figure S5 **Evolution of *let-7/mir-100* cluster and its expression in WCR**. **A**. *let-7* cluster in selected model species. Location and length information is from miRBase (version 21). Color-shaded numbers indicate the length of pre-miRNAs, and numbers with white background indicate distances between pre-miRNAs. In WCR, *dvi-miR-100* and *dvi-let-7* are located in one scaffold while *dvi-mir-125* is on a different scaffold (distance unknown). In rat and human, *let-7* cluster is separate into two and a new miRNA, *mir-99* is added. **B**. The expression pattern of miRNAs in cluster *let-7* and *mir-100* in WCR. Expression pattern was drawn based on the expression values in Supplementary Table S6. Bmo, Bombyx mori – silkworm; Ame, Apis mellifera – western honey bee; Dps, Drosophila pseudoobscura; Dme, Drosophila melanogaster – fruit fly; Tca, Tribolium castaneum – red flour beetle; Dvi, Diabrotica virgifera virgifera – western corn rootworm; Has, *homo sapiens* – human; Mmu, *Mus musculus* – house mouse; EG, egg; FI, 1st instar; SI, 2nd instar; EP, early pupa; LP, late pupa; AD, adult.
