# Supplementary figures and images for: Elucidation of the MicroRNA Transcriptome in Western Corn Rootworm Reveals Its Dynamic and Evolutionary Complexity
Source: Genomics Proteomics Bioinformatics. 2021 Feb 17;19(5):800–14. doi: 10.1016/j.gpb.2019.03.008 (PMC9170749; doi:10.1016/j.gpb.2019.03.008)

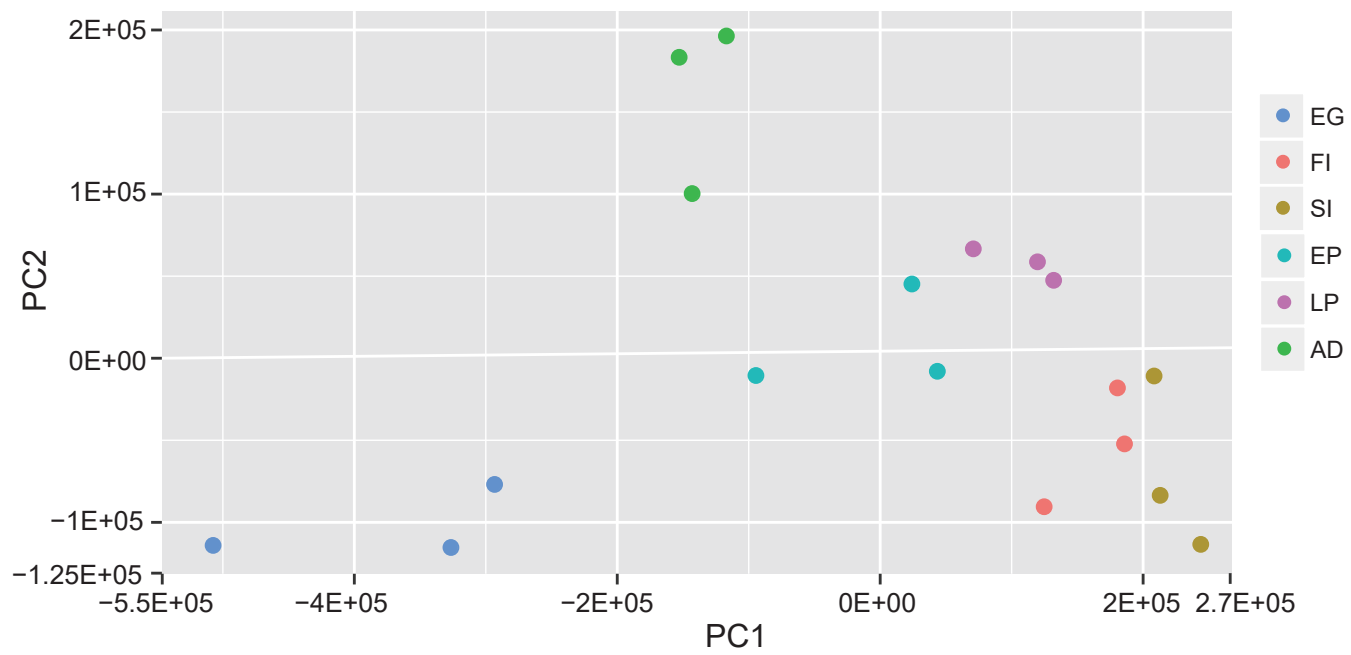

Supplement: Supplementary Figure S1 — PCA analysis of small RNA libraries from six life stages of WCR. Multidimensional scaling (MDS) analysis was performed based on the matrix of Euclidean distances and visualized using the first two components k1 and k2 with R v.3.5.1. The first dimension (73%) and second dimension (14%) were shown with X and Y axis, respectively. Samples collected from different stages were represented with different colors. PCA, principal component analysis; EG, egg; FI, 1st instar; SI, 2nd instar; EP, early pupa; LP, late pupa; AD, adult. Abbreviations similarly hereinafter. [file mmc1.pdf]

**A**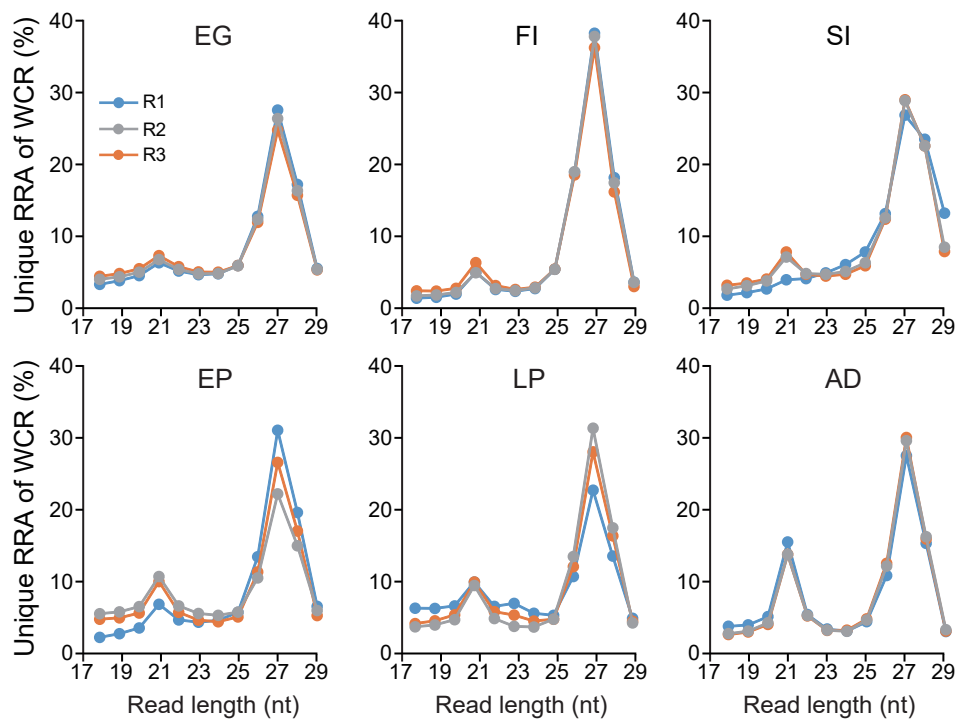**B**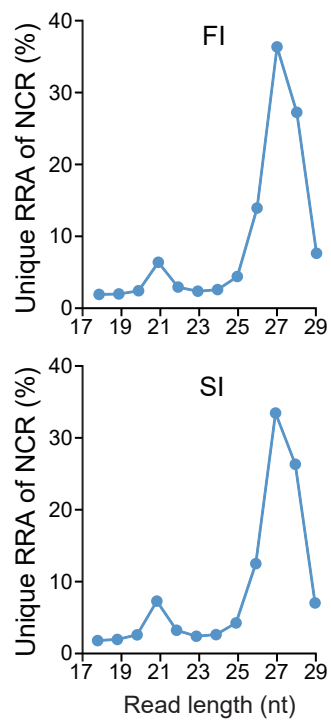

Supplement: Supplementary Figure S2 — Summary of unique reads in WCR and NCR small RNA libraries. A. Relative abundance of unique reads and length distribution in six life stages of WCR. For each life stage, there are three biological replicates. B. Unique read abundance and length distribution in 1st and 2nd instar NCR. RRA, read relative abundance; WCR, western corn rootworm; NCR, northern corn rootworm; EG, egg; FI, 1st instar; SI, 2nd instar; EP, early pupa; LP, late pupa; AD, adult. [file mmc2.pdf]

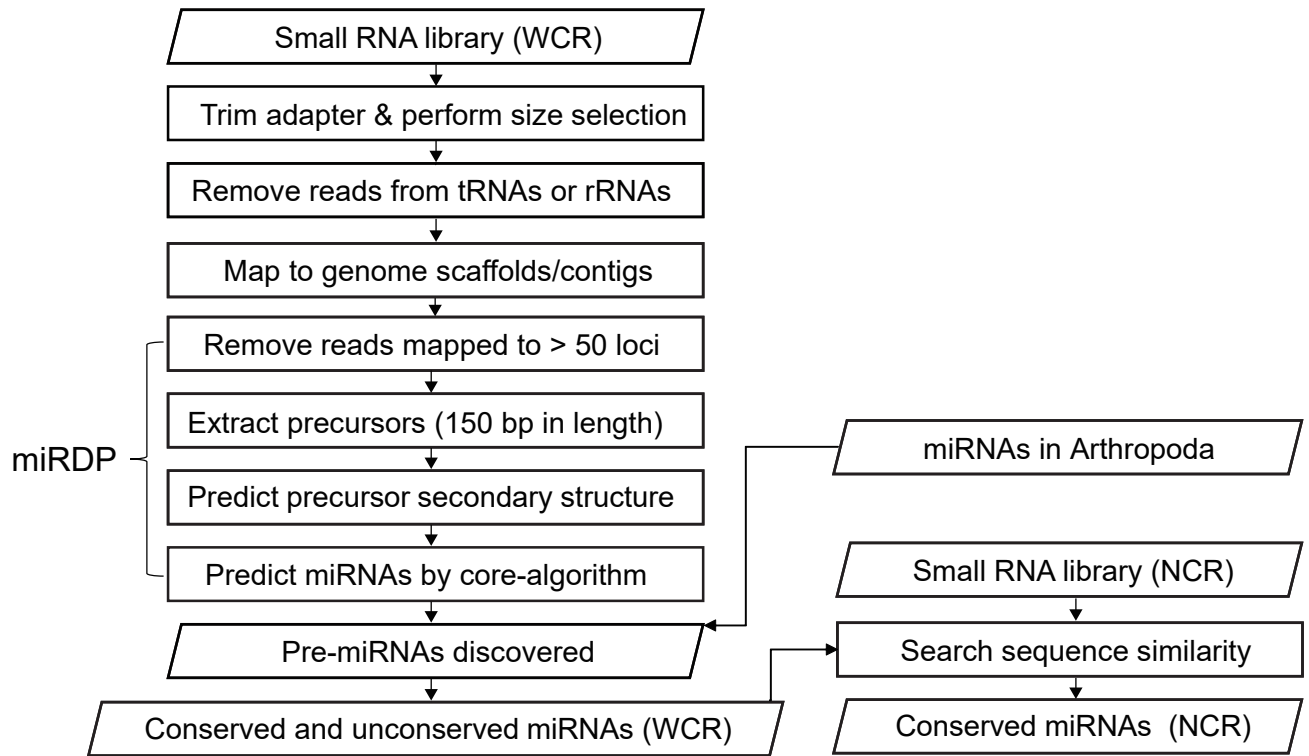

Supplement: Supplementary Figure S3 — Flowchart of identification of miRNAs in WCR and NCR. A parallelogram represents input or output while a rectangle displays a process. Sequences of tRNA and rRNA from GtRNAdb-Genomic tRNA database (Chan and Lowe 2009) and Silva-rRNA database (Quast C et al. 2013), respectively, were used to filter reads generated from tRNAs and rRNAs. miRNAs in Arthropoda are from miRBase (version 21) (Kozomara and Griffiths-Jones 2014). [file mmc3.pdf]

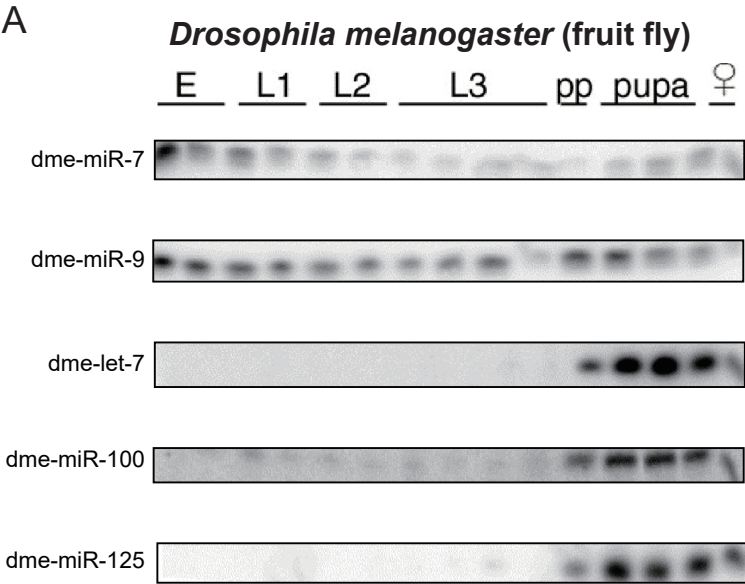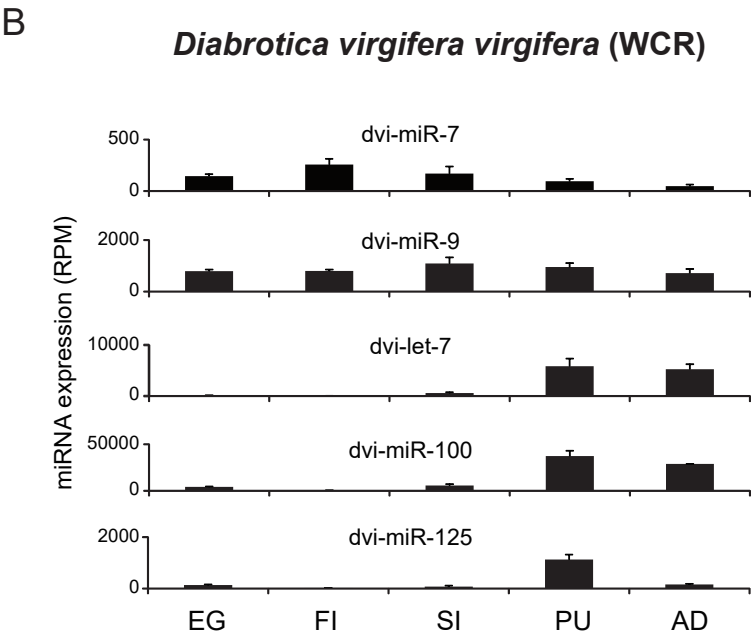

Supplement: Supplementary Figure S4 — Similarities of expression patterns of conserved miRNAs in WCR and fruit fly. A. Northern blots in fruit fly are adapted from (Sempere LF et al. 2003). Life stages in fruit fly are embryos (E), larvae (L1-L3), pre-pupae (pp), pupae (pupa), and adult females (♀), respectively. B. Expression values in WCR are from Supplementary Table S6. Values in pupae are a combination of early and late pupal stages. WCR, western corn rootworm; EG, egg; FI, 1st instar; SI, 2nd instar; PU, pupa; AD, adult. [file mmc4.pdf]

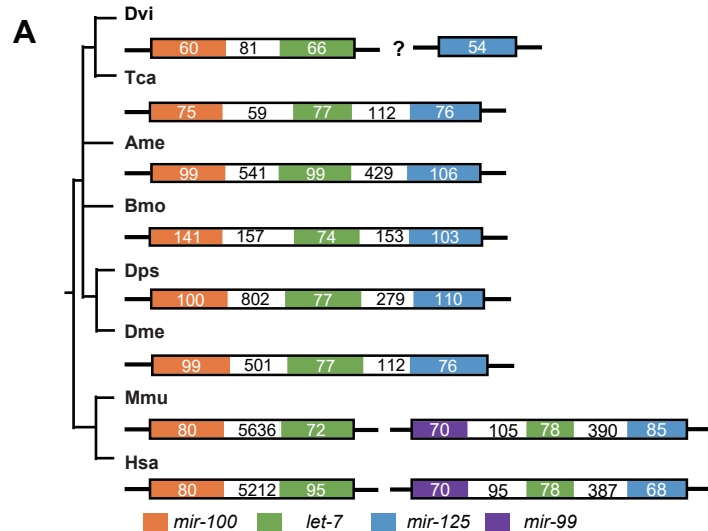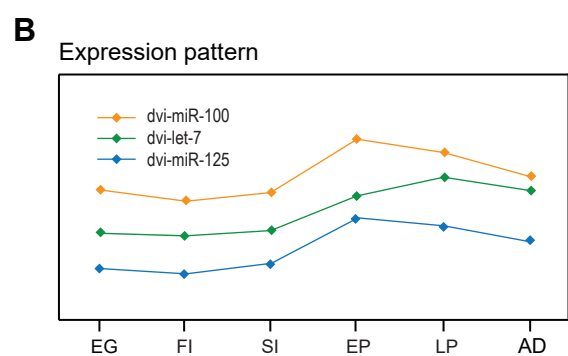

Supplement: Supplementary Figure S5 — Evolution of let-7/mir-100 cluster and its expression in WCR. A. let-7 cluster in selected model species. Location and length information is from miRBase (version 21). Color-shaded numbers indicate the length of pre-miRNAs, and numbers with white background indicate distances between pre-miRNAs. In WCR, dvi-miR-100 and dvi-let-7 are located in one scaffold while dvi-mir-125 is on a different scaffold (distance unknown). In rat and human, let-7 cluster is separate into two and a new miRNA, mir-99 is added. B. The expression pattern of miRNAs in cluster let-7 and mir-100 in WCR. Expression pattern was drawn based on the expression values in Supplementary Table S6. Bmo, Bombyx mori – silkworm; Ame, Apis mellifera – western honey bee; Dps, Drosophila pseudoobscura; Dme, Drosophila melanogaster – fruit fly; Tca, Tribolium castaneum – red flour beetle; Dvi, Diabrotica virgifera virgifera – western corn rootworm; Has, homo sapiens – human; Mmu, Mus musculus – house mouse; EG, egg; FI, 1st instar; SI, 2nd instar; EP, early pupa; LP, late pupa; AD, adult. [file mmc5.pdf]
